# Synthesis of Silver Nanoparticles Using *Odontosoria chinensis* (L.) J. Sm. and Evaluation of their Biological Potentials

**DOI:** 10.3390/ph13040066

**Published:** 2020-04-13

**Authors:** Marimuthu alias Antonysamy Johnson, Thangaiah Shibila, Santhanam Amutha, Irwin R. A. Menezes, José G. M. da Costa, Nadghia F. L. Sampaio, Henrique D. M. Coutinho

**Affiliations:** 1 Centre for Plant Biotechnology, Department of Botany, St. Xavier’s College (Autonomous), Palayamkottai, Tamil Nadu 627 002, India; ptcjohnson@gmail.com (M.a.A.J.); anletshibila@gmail.com (T.S.); 2 National Centre for Nanoscience and Nanotechnology, University of Madras, Chennai, Tamil Nadu 600025, India; amutha1994santhanam@gmail.com; 3 Department of Biological Chemistry, Centre of Biological Science and Health, Regional University of Cariri–URCA, Crato, CE 63105-000, Brazil; irwinalencar@yahoo.com.br (I.R.A.M.); galberto.martins@gmail.com (J.G.M.d.C.); nadghia.fl@gmail.com (N.F.L.S.)

**Keywords:** anti-diabetic, anti-inflammatory, cytotoxic, silver nanoparticles, *Odontosoria chinenesis*

## Abstract

The present study was aimed to synthesize silver nanoparticles (AgNPs) from the aqueous extracts of *Odontosoria chinensis* (L.) J. Sm. and the synthesized AgNPs were examined for their biopotentials. The *Odontosoria chinensis* extracts were added to 1 mM AgNO_3_ solution with different ratios viz., 0.5:9.5, 1:9, 1.5:8.5 and 2:8 ratios for the reduction of Ag ions. After reduction, the AgNPs of *Odontosoria chinensis* were analyzed spectroscopically for further confirmation. The synthesized AgNPs of *Odontosoria chinensis* were characterized by pH, ultra violet–visible spectroscopy (UV-Vis), Fourier transform–infra red spectroscopy (FT-IR), scanning electron microscopy-energy dispersive X-ray analysis (SEM-EDAX) and X-Ray diffraction (XRD). The time taken for the complete reduction of Silver (Ag) in solution to nanoparticle was 10 min. The *O. chinensis* aqueous extracts mediated silver nanoparticles showed a broad peak with distinct absorption at around 400–420 nm and confirmed the silver nanoparticle formation. FT-IR results also confirmed the existence of organic materials in the silver nanoparticles of *O. chinensis*. The EDX spectra of AgNPs of *O. chinenesis* revealed the occurrence of a strong Ag peak. The synthesis of AgNPs of *O. chinenesis* was confirmed with the existence of a peak at 46.228°. The toxic potential of AgNPs of *O. chinenesis* showed varied percentage mortality with the LC_50_ values of 134.68 μL/ 50 mL and 76.5 μL/50 mL, respectively. The anti-inflammatory and anti-diabetic activities of aqueous and AgNPs of *O. chinenesis* were statistically significant at *p* < 0.05 level. Conclusion: The results demonstrated the toxicity, anti-diabetic and anti-inflammatory potential of the studied AgNPs. The synthesized nanoparticles of *Odontosoria chinensis* could be tested as an alternative to anticancer, anti-diabetic and anti-inflammatory drugs.

## 1. Introduction

*Odontosoria chinensis* (L.) J. Sm. (Syn. *Trichomanes chinensis* L. and *Sphenomeris chinensis* (L.) Maxon) belongs to the family Lindsaeaceae and is a terrestrial herb found at higher altitudes [1]. The plant is used in the treatment of chronic enteritis [2,3]. Toji [4] identified the existence of flavonoids, phenols and steroids in the acetone and methanolic extracts of *Odontosoria chinensis*. In addition, Toji [4] also observed the antibacterial activity of *Odontosoria chinensis* acetone extracts against *Pseudomonas aeruginosa* and *Staphylococcus aureus*. Methanolic and ethyl acetate extracts fraction showed significant antioxidant activities as well as antimicrobial activity against *Escherichia coli.* Two major secondary metabolites 3,4-dihydroxybenzoic acid and 3,4-dihydroxybenzaldehyde were isolated from *T. chinensis* leave, stem and root methanolic extracts [5]. Four aromatic compounds viz., 3,4 dihydrozybenzoic acid, 3,4-dihydroxybenzaldehyde, 4-hydroxy-3, 5-dimethoxybenzoic acid and 4-hydroxy-3-methoxy benzoic acid were isolated from *T. chinensis*. Al-Mekhlafi [6] studied the cytotoxic and acetylcholinesterase inhibitory activities of *Odontosoria chinensis* crude fractions. The whole plants of *Odontosoria chinensis* was used to treat itches [7].

Nowadays, silver nanoparticles have gained tremendous popularity at the global level in the field of agriculture, medicine, sensor, and pharmaceutical industries. Generally, two methods are used for the synthesis of silver nanoparticles viz., biological and chemical. Due to negative impacts on the ecosystem, time consumption and production cost, the chemical methods are not popularized [8]. On the other hand, biosynthesis of silver nanoparticles has attained its own importance due to time consumption for synthesis, and being economically cheap and more eco-friendly. For the last two decades, many researchers focused their attention to synthesize silver nanoparticles using natural products as the source [9,10,11,12,13,14,15,16]. With reference to pteridophytes, only very few reports are available for the green synthesis of AgNPs [17,18,19]. However, there is no report on *Odontosoria chinensis* (L.) J. Sm. mediated silver nanoparticle synthesis. Hence, the present study aimed to synthesize AgNPs from the aqueous extracts of *Odontosoria chinensis* (L.) J. Sm. and examine their anti-inflammatory, anti-diabetic and toxic effects.

## 2. Materials and Methods

Healthy and disease-free matured sporophytes without spores of *Odontosoria chinensis* (L.) J. Sm. (Indigenous to Hawai, Phillipines and other parts of the tropics) were collected from Kodaikanal Botanical Garden, Eetipallam, Kodaikanal, Tamil Nadu, India. The collected sporophytes were washed in running tap water to remove the unwanted debris materials. The plant materials were dried and the excess water was removed using blotting paper.

A total of 10 g of matured sporophytes of *O. chinensis* without spores were cut into small pieces and boiled with 100 mL of distilled water for 30 min. After 30 min, the aqueous extracts were filtered using Whatman No. 1 filter paper. The filtered extracts were centrifuged at 3000 rpm for 10 min. The supernatants were collected and used for synthesis of AgNPs and further studies [16]. Qualitative analysis of the aqueous extracts was carried out according to the standard method described by Harborne [20]. The effects of AgNO_3_ concentration (1mM–6mM), different time intervals for the reduction and various temperatures (30–90 °C) on the synthesis of AgNPs of *Odontosoria chinensis* were studied.

The *Odontosoria chinensis* extracts were added to 1 mM AgNO_3_ solution with different ratio viz., 0.5:9.5, 1:9, 1.5:8.5 and 2:8 ratios for the reduction of Ag ions. After reduction, the AgNPs of *Odontosoria chinensis* were analyzed spectroscopically for further confirmation. The reduction of pure Ag ions was monitored by measuring the ultra violet–visible spectroscopy (UV-Vis) spectrum of the solution at 400–900 nm using a Shimadzu spectrophotometer and the characteristic peaks were detected. The AgNPs and *Odontosoria chinensis* aqueous extracts were pelletized separately in IR-grade KBr (Hi Media, Mumbai, India). FT-IR (Fourier-transform infrared spectroscopy) analysis was performed using a Perkin Elmer Spectrophotometer system (PerkinElmer, Waltham, MA, USA) over a frequency range 400–4000 cm^−1^, which was used to detect the characteristic peaks. Each and every analysis was repeated twice for the confirmation of the spectrum. The synthesized AgNPs of *Odontosoria chinensis* were characterized by pH and X-Ray Diffraction (XRD). Morphology and size of AgNPs were investigated by the FESEM (FEI-TECHNAI G2, Tokyo, Japan) the resolution was 1.0 nm/15 KV (1.6 nm/1 KV).

### 2.1. Toxicity Analysis

The toxicity of synthesized silver nanoparticles of *Odontosoria chinensis* was determined using *Artemia salina* (naupli) as the experimental animal [21]. Aqueous extracts and nanosolutions of *Odontosoria chinensis* AgNPs were taken in different concentrations (50, 100, 150, 200 and 250 μL/50 mL) and distilled water was used as the control. For each and every experimental concentration, 10 naupli were employed. After 24 h, the mortality of the naupli was observed. Each and every experiment was repeated thrice with five replicates. LC_50_ at 95% confidence limit, LCL—Lower Confidence Limit and UCL—Upper Confidence Limit were calculated. The plumbagin was used as standard to estimate the toxicity.

### 2.2. Anti-Diabetic Activity

The aqueous and silver nanoparticles of *Odontosoria chinensis* were tested for its serum amylase inhibitory activity by the Yukihiko method [22]. Three test tubes were taken and labeled as blank (B), test (T) and control (C). Then, 2.5mL of phosphate buffer with pH 6.8 was added to each tube. The substrate starch (1mL) and sodium chloride were added to all the prepared aliquots. The test tubes were incubated at 37 °C for 10 min. After incubation, 0.5mL of aqueous extract of *O. chinensis* was added and 0.2 mL of the enzyme (serum, amylase, pancreatic amylase, α-amylase from fermented barley) was added to the test tube. The contents of the test tube were mixed well and incubated at 37 °C for 10 min. After incubation, 0.5mL of 2N NaOH was added to the test tube (T) and (C), 0.2 mL of enzymes was added to the control (C), 5.7 mL of distilled water alone served as the blank (B), and 0.2 mL of dinitrosalicylic acid was added to all the test tubes (T and C). The contents were mixed well and kept in a boiling water bath for 15 min. The standard acarbose was employed as a positive control. The intensity of reddish orange color was read at 540 nm. The percentage of inhibitory action was calculated using the following formula.

Percentage inhibition = Optical Density (O.D) of control − O.D of test / O.D of control × 100(1)

### 2.3. Anti-Inflammatory Activity using Membrane Stabilization Assay

#### 2.3.1. Preparation of Red Blood Cells (RBCs) Suspension

The blood was collected from a healthy human volunteer who had not taken any NSAIDs (non steroidal anti-inflammatory drugs) for 2 weeks prior to the experiment and transferred to the centrifuge tubes and centrifuged at 3000 rpm for 10 min. The tubes were washed thrice with equal volume of normal saline. The volume of blood was measured and reconstituted as 10% *v/v* suspension with normal saline [23,24].

#### 2.3.2. Heat Induced Haemolysis

The reaction mixture (2 mL) includes 1 mL of different concentrations of aqueous and silver nanoparticles of *Odontosoria chinensis* (50, 100, 200 and 250 µg/mL) and 1 mL of 10% RBCs suspension. For the negative control, the saline was added instead of plant extracts. Aspirin was employed as a positive control. The reaction mixtures were incubated in a water bath at 56 °C for 30 min. After incubation, the tubes were kept in running tap water. The reaction mixtures were centrifuged at 3000 rpm for 5 min and the absorbance of the supernatants was measured at 560 nm [25]. The experiment was performed in triplicate for all the test samples. As a standard, 100 µg/mL of Indomethacin was used.

The oercentage inhibition of haemolysis was calculated as follows:

Percentage inhibition = (Abs control − Abs sample) / Abs control × 100(2)

Statistical analysis was performed using SPSS 21 software. Analysis of variance and pair wise Pearson and Spearsman correlation tests were performed. The *p* value < 0.05 was considered as significant.

## 3. Results

### 3.1. Synthesis of O. Chinensis AgNPs

Among the various ionic concentrations of AgNO_3_ tested, the stable nanoparticle of *Odontosoria chinensis* was obtained at 1 mM (1:9 ratios) concentration of AgNO_3_. Among the various temperatures examined for the synthesis of silver nanoparticles of *O. chinensis*, the nanoparticle synthesized at 40 ˚C showed small size of nanoparticles (22.3–48.2 nm). The silver nanoparticles synthesised above 40 °C demonstrated the occurrence of agglomeration of AgNPs. Green synthesized AgNPs of *O. chinensis* were stable for 6 months without shifting the surface plasmon absorbance band and free from microbial contamination. AgNO_3_ solution mixing with aqueous extracts of *O. chinensis* showed that the color of the solution changed from colorless or pale yellow to a yellowish brown color and indicated the presence of AgNPs. The time taken for the complete reduction of Ag in solution to nanoparticle was 10 min. After 2 h of incubation, no further increase in color intensity was found in the AgNPs of *O. chinensis*, indicating the complete reduction of silver ions. The pH of the AgNO_3_ solution was also changed from 3.87 to 4.56 when the AgNO_3_ solution was mixed with *O. chinensis* aqueous extracts. The change of pH suggested the capping between Ag and *O. chinensis* aqueous extracts.

### 3.2. Spectroscopic Analysis of O. Chinensis AgNPs

**Figure 1:**
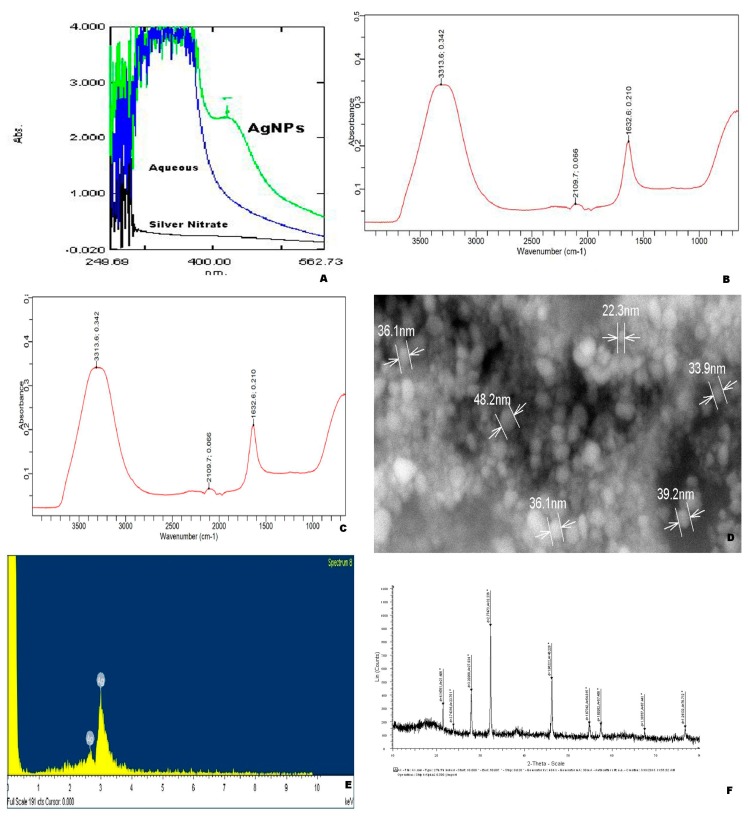
Biosynthesis of silver nanoparticles (AgNPs) of *O. chinensis*; UV-Vis spectrum of aqueous, silver nitrate and silver nanoparticles of *O. chinensis (***A)**; FT-IR spectrum of *O. chinensis* aqueous extracts (**B**); Fourier-transform infrared (FT-IR) spectrum of silver nanoparticles of *O. chinensis* (**C**); scanning electron microscopy (SEM) photograph of silver nanoparticles of *O. chinensis* (**D**); energy dispersive X-ray analysis (EDAX) spectrum of *O. chinensis* silver nanoparticles (**E**); X-Ray diffraction (XRD) pattern of *O. chinensis* silver nanoparticles (**F**)

The absorption peak (SPR) was observed in the visible range at 405 nm. UV-Vis spectra of *O. chinensis* aqueous extracts failed to display an absorption in the range of 400–800 nm but the *O. chinensis* aqueous extracts mediated silver nanoparticles showed a broad peak with distinct absorption at around 400–420 nm and confirmed the silver nanoparticle formation (Figure 1A). In addition, the silver nanoparticles of *O. chinensis* displayed a peak at 660 nm and confirmed the existence of some phytoconstituents.

In the present study, FT-IR was used to identify the chemical composition of surface, local molecular environment, reducing and capping agents of synthesized silver nanoparticles of *O. chinensis*. The results of FT-IR analysis of *O. chinensis* silver nanoparticles showed different stretches of bonds with varied peaks (Figure 1B,C). The peak, 3317.3 cm^−1^, was assigned to O – H stretch in the reducing agent (phenol), 2109.7 cm^−1^ to the CH stretch in alkane and 1632.6 cm^−1^ to C–C stretch (in-ring) from carbonyl stretch in aromatics. Fourier transforms infra radiation (FTIR) spectroscopic peak profiles were used to confirm the presence of carboxylic acid on the green synthesized silver nanoparticles. This study results also confirmed the existence of organic materials in the silver nanoparticles of *O. chinensis*. These FT-IR spectroscopic study results confirmed that *O. chinensis* aqueous extracts possess the capacity to perform dual functions of reduction and stabilization of silver nanoparticles.

### 3.3. SEM-EDAX Analysis of O. Chinensis AgNPs

The morphological characters of synthesized AgNPs of *O. chinensis* were determined using a scanning electron microscope (Figure 1D). The size of *O. chinensis* AgNP was observed in the range of 22.3–48.2 nm. The average size of the nanoparticle is 35.97 ± 8.38 nm. However, further observations with higher magnification reveal that these crowded AgNPs are groups of smaller nanoparticles that exhibit good uniformity. The spherical and oval shaped AgNPs of *O. chinensis* was observed (Figure 1D). The SEM-EDAX confirmed the existence of silver in the AgNPs of *O. chinensis* (Figure 1E).

### 3.4. XRD Analysis of O. Chinensis AgNPs

The XRD spectra of AgNPs of *O. chinensis* revealed the occurrence of a strong Ag peak. The XRD pattern of *O. chinensis* nanoparticles illustrated 9 peaks at 21.406°, 23.751°, 27.834°, 32.236°, 46.228°, 54.815°, 57.456°, 67.441° and 76.712° (Figure 1F). The synthesis of AgNP’s of *O. chinensis* confirmed with the existence of a peak at 46.228°.

### 3.5. Biopotency of O. Chinensis AgNPs

**Figure 2:**
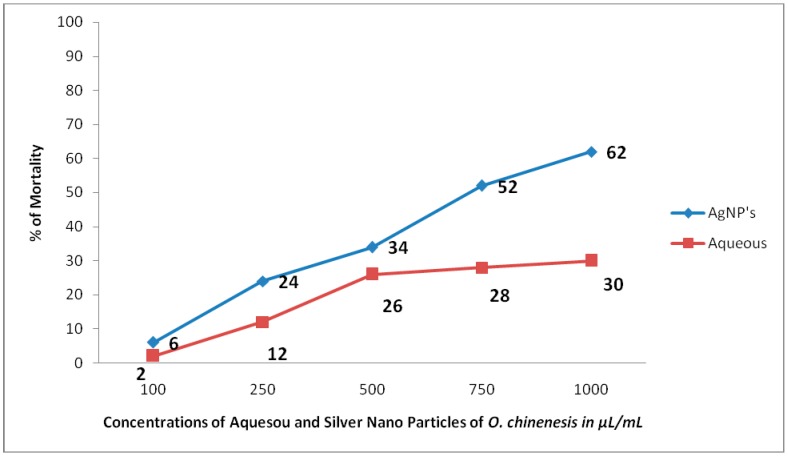
Toxic potential of the aqueous extracts and AgNPs of *O. chinensis*.

**Figure 3:**
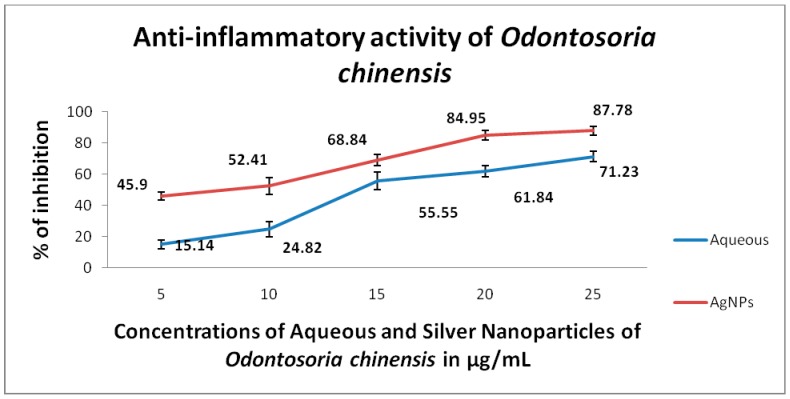
Anti-inflammatory activity of the aqueous extracts and AgNPs of *O. chinensis*.

**Figure 4:**
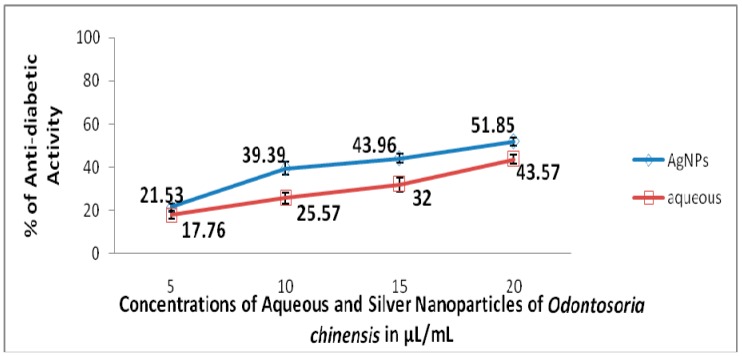
Anti-diabetic activity of the aqueous extracts and AgNPs of *O. chinensis*.

The toxic potential of the aqueous extracts and AgNPs of *O. chinensis* showed varied percentage mortality with the LC_50_ values of 134.68 μL/50 mL = 2.69 µL/mL and 76.48 μL/50 mL = 1.53 µL/mL, respectively. A dose dependent toxicity was observed in AgNPs of *O. chinensis* (Figure 2). The standard plumbagin showed 100% mortality of brine shrimp nauplii at 0.046 mg/mL. Similar to toxicity, the dose dependent anti-inflammatory and anti-diabetic activities were observed in the aqueous and AgNPs’ of *O. chinensis* ( Figure 3; Figure 4). The AgNPs of *O. chinensis* showed to be more anti-inflammatory and inhibited the specific enzymes responsible for anti-diabetic activities than the aqueous extracts of *O. chinensis* (Figure 3 and Figure 4). The anti-inflammatory and anti-diabetic activities of aqueous and AgNPs’ of *O. chinensis* were statistically significant at *p* < 0.05 level. The standard indomethacin (100 µg/mL) showed 71.43% inhibition. The standard acarbose 500 µg/mL showed 78% activity. A strong positive correlation was observed with various concentrations of aqueous extracts and anti-inflammatory activity of *O. chinensis* with the correlation coefficent R = 0.972 and silver nanoparticles of *O. chinensis* with the correlation coefficent R = 0.965. The correlation was significant at the 0.05 level (2-tailed). A strong positive correlation was observed with various concentrations of aqueous extracts and anti-diabetic activity of *O. chinensis* with the correlation coefficent R = 0.983 and silver nanoparticles of *O. chinensis* with the correlation coefficent R = 0.78. The correlation was significant at the 0.05 level (2-tailed).

## 4. Discussion

Generally, 1 mM AgNO_3_ (1:9 ratio) was employed for the synthesis of silver nanoparticles from Pteridophytes [16,17,18,19]. The nanoparticles were stable up to six months without any contamination. In the present study, 1 mM of silver nitrate also produced good nanoparticles of *O. chinensis*. Similar results were observed in *Cyathea nilgirensis* in our previous research, carried out in our lab. Among the various ratios of *O. chinensis* extracts with 1 mM AgNO_3_ screened, the 1:10 ratio was optimized for silver nanoparticle synthesis. Bhor et al. [24], Nalwade et al. [18], Sant et al. [26] and Johnson et al. [16] employed 1:10 silver nitrate and plant extracts ratio for the synthesis of silver nanoparticles from *Nephrolepis exaltata, Cheilanthes farinosa, Adiantum phillippense* and *Cyathea nilgirensis*. In the present study, 10 mL of *O. chinensis* and 90 mL of 1mM silver nitrate also yielded good nanoparticles. Commonly, the UV-Vis spectroscopy was employed to confirm the nanoparticle formation [27]. Feldheim and Foss [28] suggested the light wave length ranges i.e., 300 to 800 nm for characterizing various nanoparticles. Huang et al. [29] further confirmed the spectroscopic measurement for nanoparticles at 400–450 nm. Shivakumar and Vidyasagar [30] and Christopher et al. [31] observed a characteristic peak for silver nanoparticle of at 420 nm for *Annona reticulata* and *Aegle marmelos*, respectively. In the present study, a characteristic peak was also observed at 405 nm for the silver nanoparticles of *O. chinensis*. These UV-Vis analysis results suggest that the phytoconstituents that occurred in the aqueous extracts of *O. chinensis* may be responsible for the reduction. Preliminary phytochemical analysis of *O. chinensis* aqueous extracts confirmed the presence of terpenoids, tannins, coumarins, phenolics and steroids. These metabolites are responsible for the bio-reduction of AgNPs. Earlier studies indicated that the reduction of silver ions and stability of AgNPs are due to the occurrence of phytoconstituents or metabolites present in the source extracts [32]. To probe the chemical composition of the surface and the local molecular environment, reducing and capping agents of the silver nanoparticles, FT-IR spectroscopy was employed [16]. In the present study, reduction and capping of the silver nanoparticles of *Odontosoria chinensis* was also confirmed by the FT-IR analysis.

Brine shrimp bioassay was employed to determine the different pharmacological properties of the plant extracts [33]. In this study, toxic effects of *Odontosoria chinensis* nanoparticles towards shrimp’s larvae were studied. A dose dependent toxicity was observed in AgNPs of *O. chinensis*. The AgNPs of *O. chinensis* showed more toxic activities than the aqueous extracts of *O. Chinensis*. The toxic effects of *O. chinensis* nanoparticles can be correlated with anticancer activity of the *O. chinensis* nanoparticles. The results of the present study directly coincide with Johnson et al.’s [16] observation on AgNPs of *C. nilgirensis*. The results of the present study suggested that *O. chinensis* AgNPs treatment against *Artemia salina* inhibited the viability. In control experiments, it was clearly indicated that all the concentrations of aqueous and AgNPs extracts did not induce any lethal effect on *A. salina*. We observed the morphological changes, which disrupted and affected movement and feeding, which resulted mainly in decreased swimming ability. Intestinal enlargement, loss of antennae and deformation of antennae occured in *A. salina* exposed to *O. chinensis* aqueous extract and AgNPs of *O. chinensis*. However, we failed to observe the significant changes in *A. salina* cultured in the control (saline water). From the results, the percentage of mortality rate was extensively increased, which corresponds to the concentration of aqueous and *O. chinensis* AgNPs. The exposure of silver nanoparticles of *O. chinensis* may generate the reactive oxygen species (ROS) that may cause cytotoxicity [34]. The synthesized nanoparticles of *Odontosoria chinensis* could be an alternative source of anticancer drugs [35].

Preliminary phytochemical analysis of *O. chinensis* aqueous extracts confirmed the presence of terpenoids, tannins, coumarins, phenolics and steroids. The FT-IR analysis results confirmed the occurrence of polyphenols/phenols in the silver nanoparticles of *O. chinensis*. The occurrence of tannins, phenolics and terpenoids are responsible for the anti-inflammatory and anti-diabetic activities of the silver nanoparticles and aqueous extracts of *O. chinensis*. The tannins prevent hyperglycemia by inhibiting aldose reductase in vitro and induced lens pacification on organ culture. Tannins also inhibit sorbitol formation in the lens, and might counter the polyol pathway induced oxidative stress. Suryanarayana [36] reported the anti-diabetic properties of tannoids. The high phenolic content of the aqueous extracts of the *O. chinensis* support the anti-amylase activity. The phenolic substances have the ability to interact with and/or inhibit proteins/enzymes [37]. The Spearman and Pearson correlation results clearly explained the dose dependent protection of the studied aqueous and AgNPs, with 0.982 of *O. chinensis*, and correlation was significant at the 0.01 level (2-tailed). Similar to our observation, Shaik et al. [38] also observed the highest percentage of antibacterial activity in the silver nanoparticles of *Origanum vulgare* compared with aqueous extracts of *Origanum vulgare.* The *Origanum vulgare* extract failed to display any microbicidal activity up to 300 µg, but the AgNPs prepared from *Origanum vulgare* displayed microbicidal activity. Similarly, the viability of tumor cells treated with AgNPs were greatly affected (88 ± 4.44% cell death) compared with untreated cells (2% cell death) after 24 h of incubation [11].

## 5. Conclusions

The results of the present study clearly explain the toxicity, anti-diabetic and anti-inflammatory potentials of the studied silver nanoparticles of *Odontosoria chinensis.* The synthesized nanoparticles of *Odontosoria chinensis* could be tested as an alternative source of anticancer, anti-diabetic and anti-inflammatory drugs.
